# Supplementary material for: A projection specific logic to sampling visual inputs in mouse superior colliculus
Source: eLife. 2019 Nov 21;8:e50697. doi: 10.7554/eLife.50697 (PMC6872211; doi:10.7554/eLife.50697)

Figure 2 – Figure Supplement 1. All 599 cells (445 LP; 263 Pbg) in their corresponding cluster

A Cluster 1 | 1wt | sOFFα

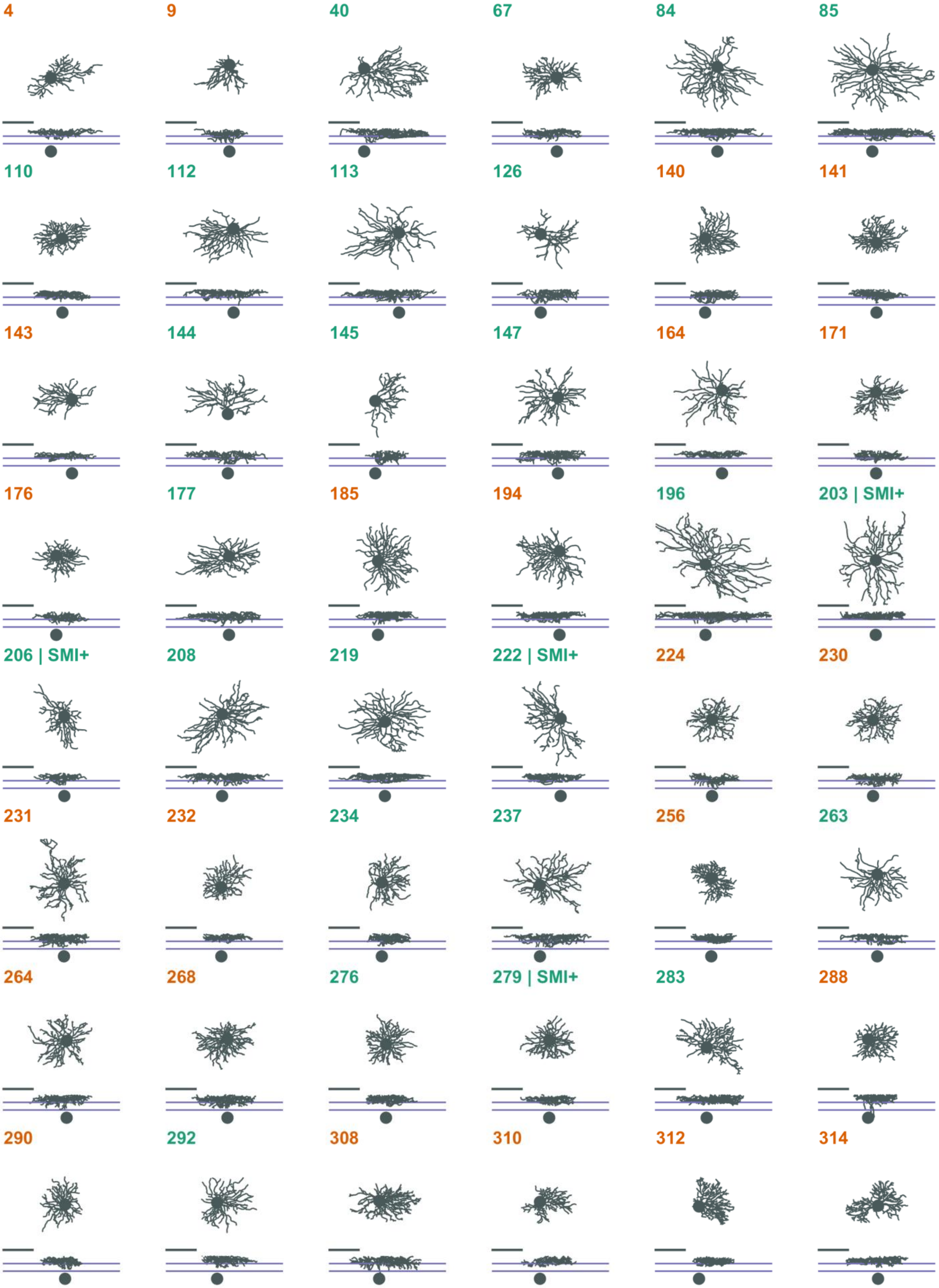

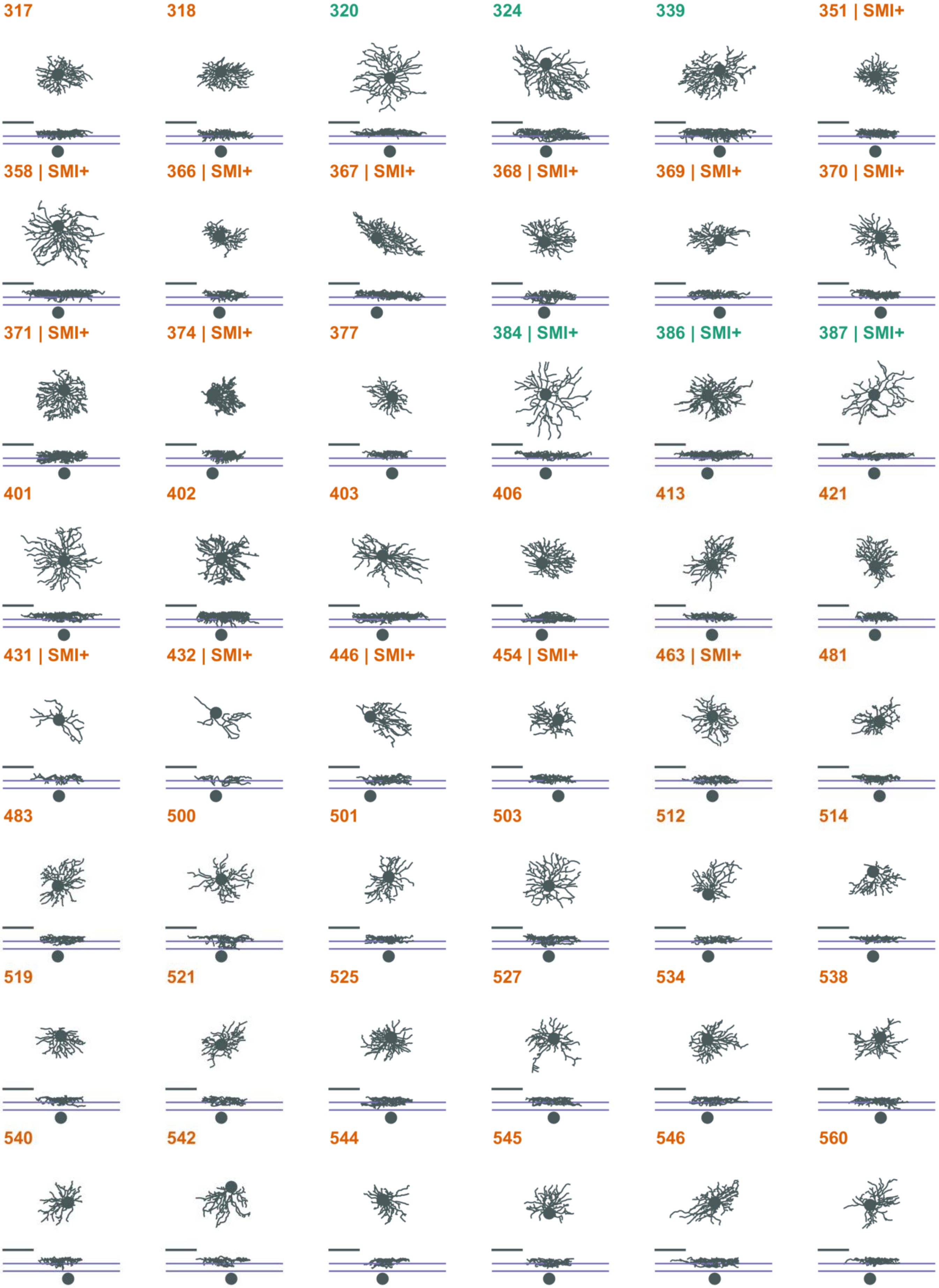
317
318
320
324
339
351 | SMI+
358 | SMI+
366 | SMI+
367 | SMI+
368 | SMI+
369 | SMI+
370 | SMI+
371 | SMI+
374 | SMI+
377
384 | SMI+
386 | SMI+
387 | SMI+
401
402
403
406
413
421
431 | SMI+
432 | SMI+
446 | SMI+
454 | SMI+
463 | SMI+
481
483
500
501
503
512
514
519
521
525
527
534
538
540
542
544
545
546
560

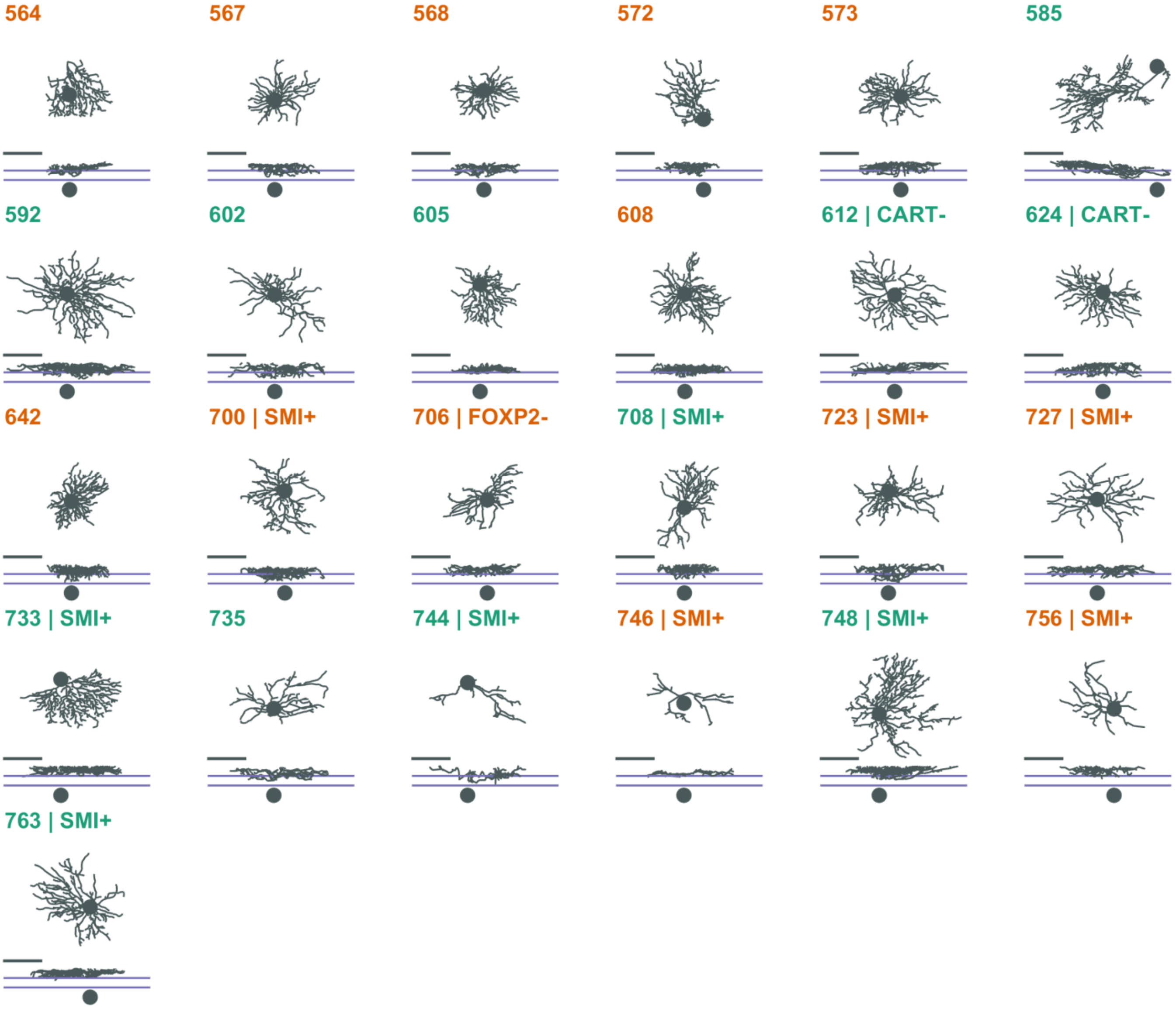
564
567
568
572
573
585
592
602
605
608
612 | CART-
624 | CART-
642
700 | SMI+
706 | FOXP2-
708 | SMI+
723 | SMI+
727 | SMI+
733 | SMI+
735
744 | SMI+
746 | SMI+
748 | SMI+
756 | SMI+
763 | SMI+

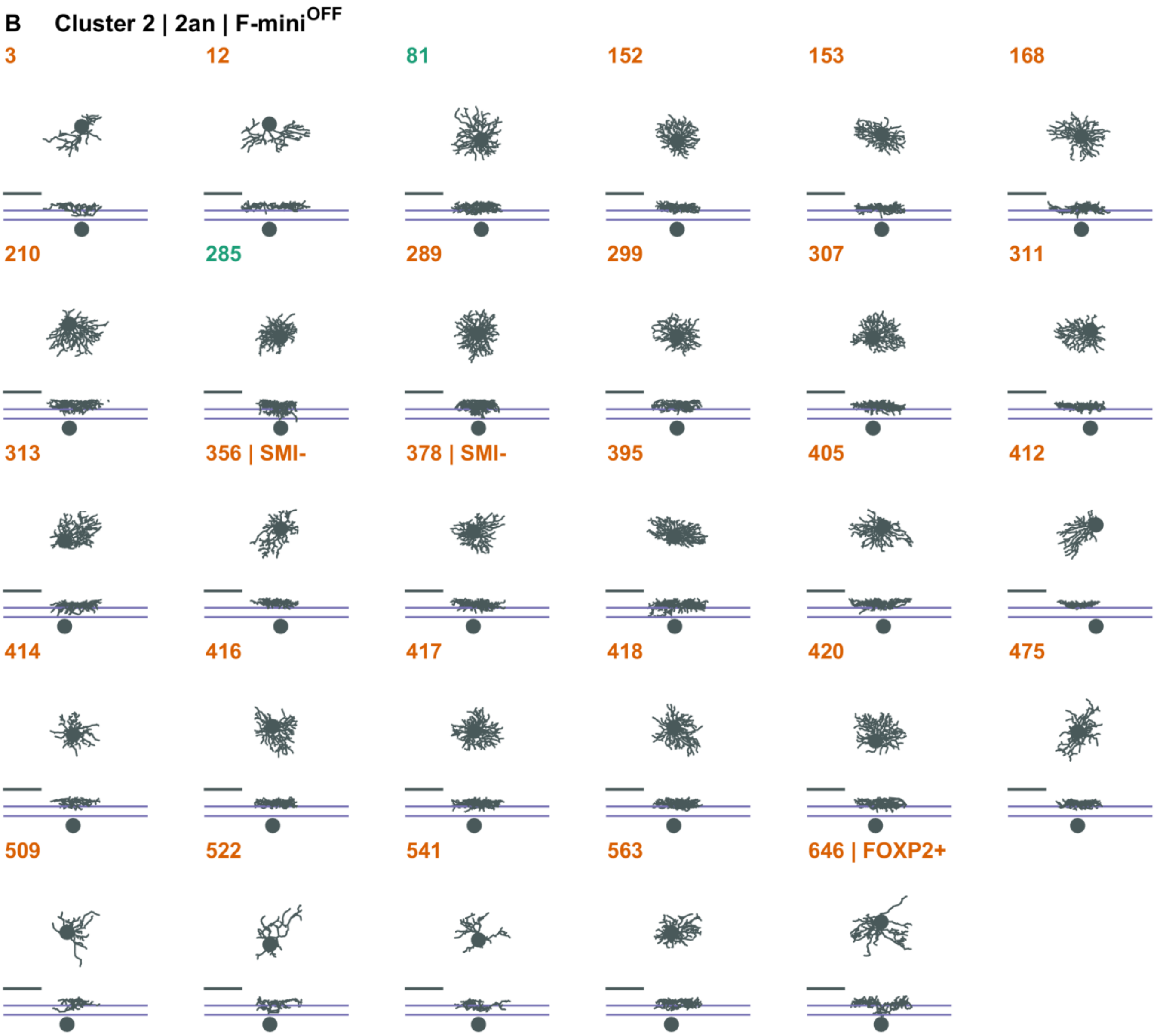
B
Cluster 2 | 2an | F-mini OFF
3
12
81
152
153
168
210
285
289
299
307
311
313
356 | SMI-
378 | SMI-
395
405
412
414
416
417
418
420
475
509
522
541
563
646 | FOXP2+

C Cluster 3 | 27

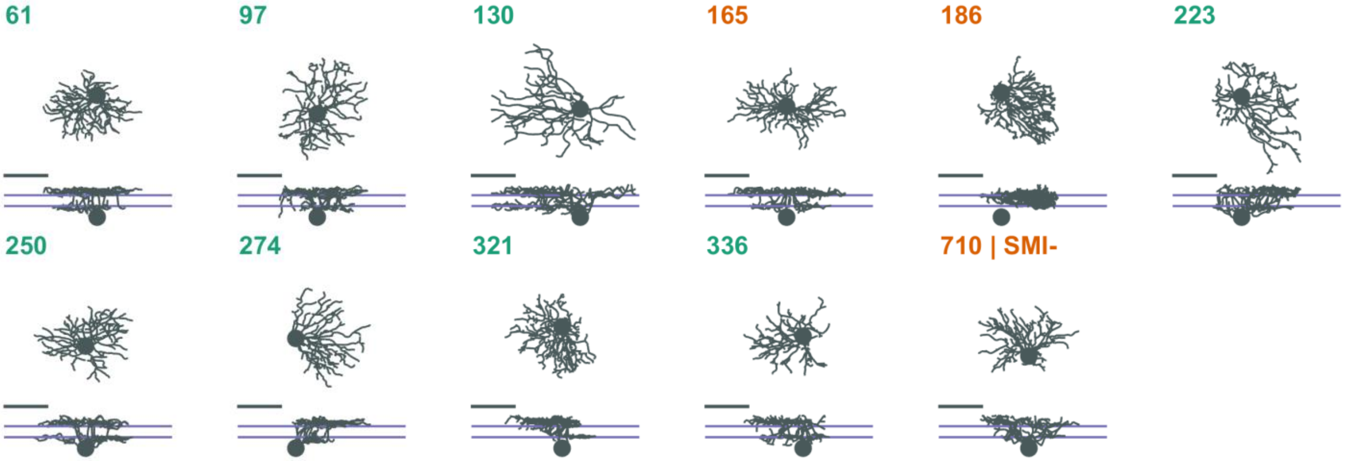

D Cluster 4 | 37c | ON-OFF DS

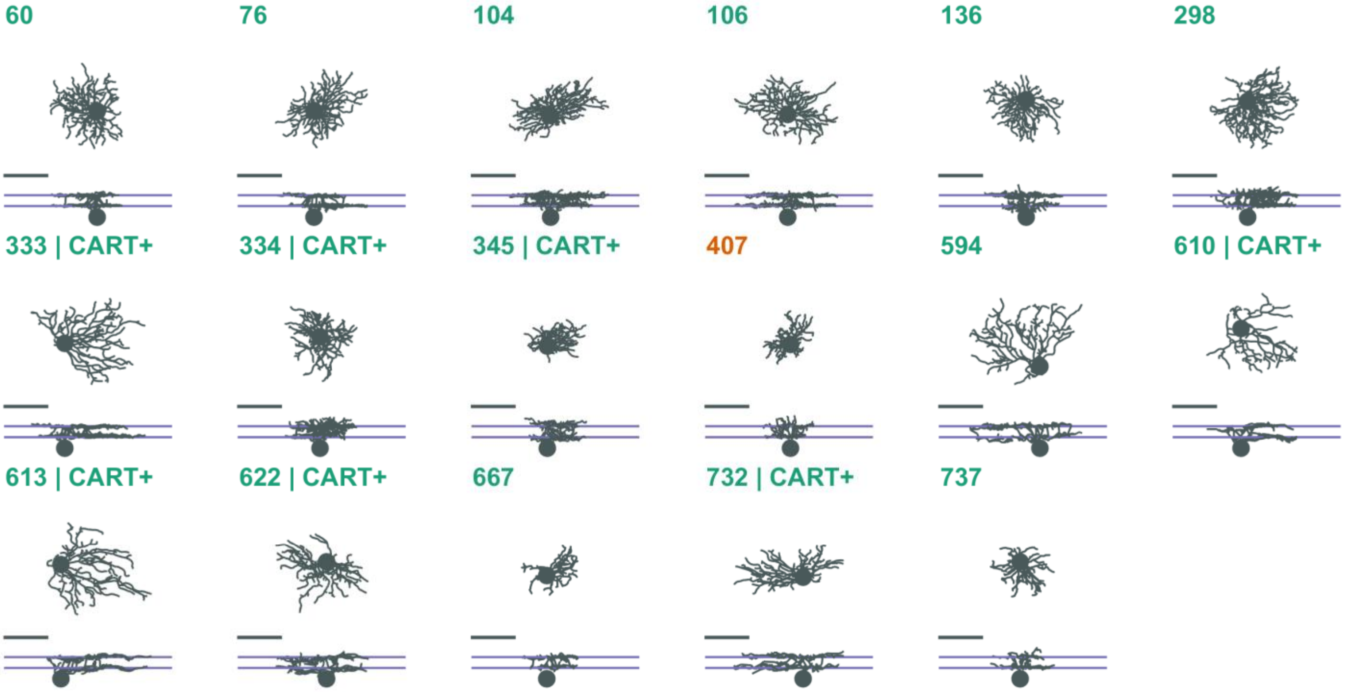

E Cluster 5 | 4i/4on | mini tOFFα

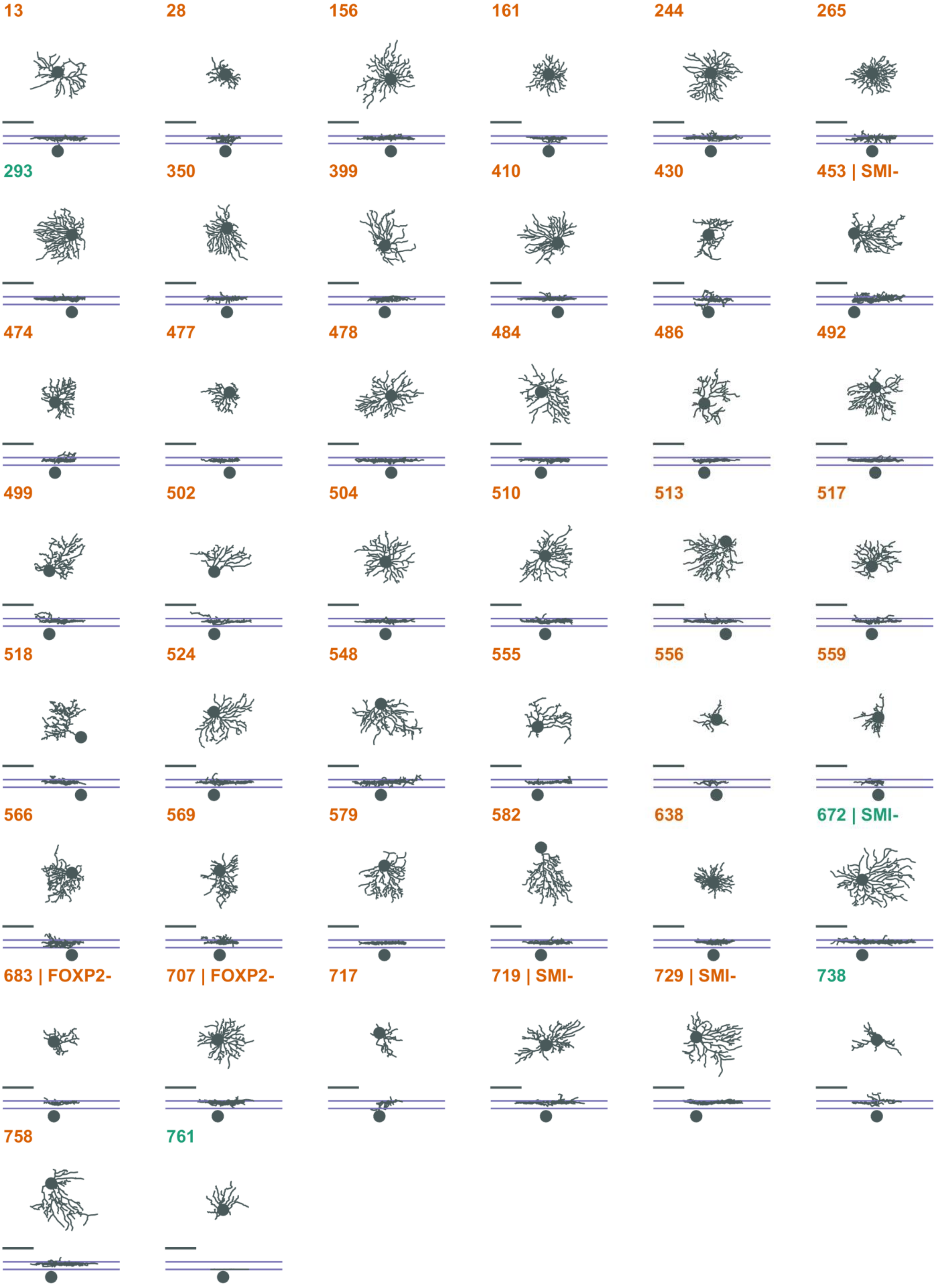

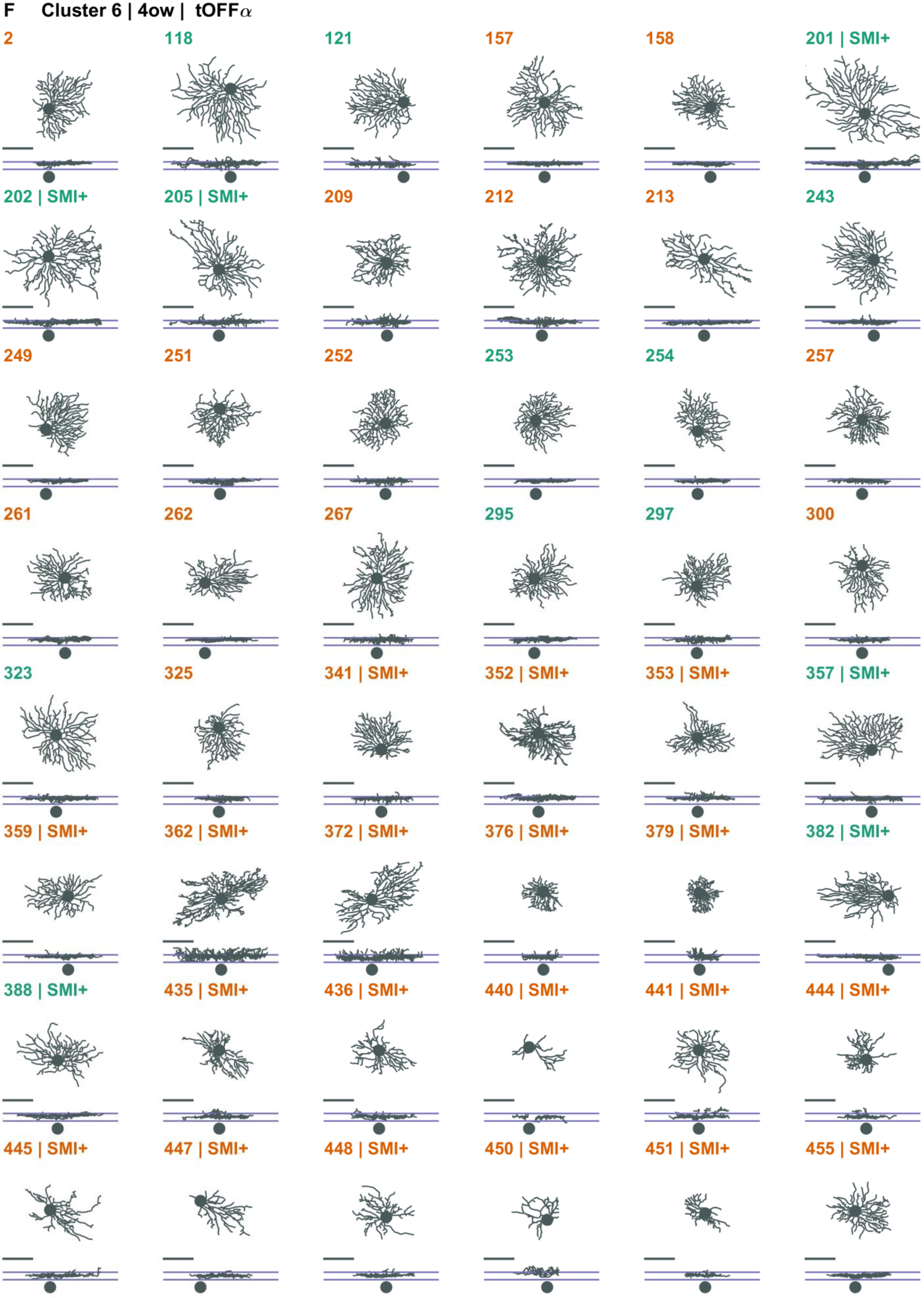
F
Cluster 6 | 4ow | tOFFα
2
118
121
157
158
201 | SMI+
202 | SMI+
205 | SMI+
209
212
213
243
249
251
252
253
254
257
261
262
267
295
297
300
323
325
341 | SMI+
352 | SMI+
353 | SMI+
357 | SMI+
359 | SMI+
362 | SMI+
372 | SMI+
376 | SMI+
379 | SMI+
382 | SMI+
388 | SMI+
435 | SMI+
436 | SMI+
440 | SMI+
441 | SMI+
444 | SMI+
445 | SMI+
447 | SMI+
448 | SMI+
450 | SMI+
451 | SMI+
455 | SMI+

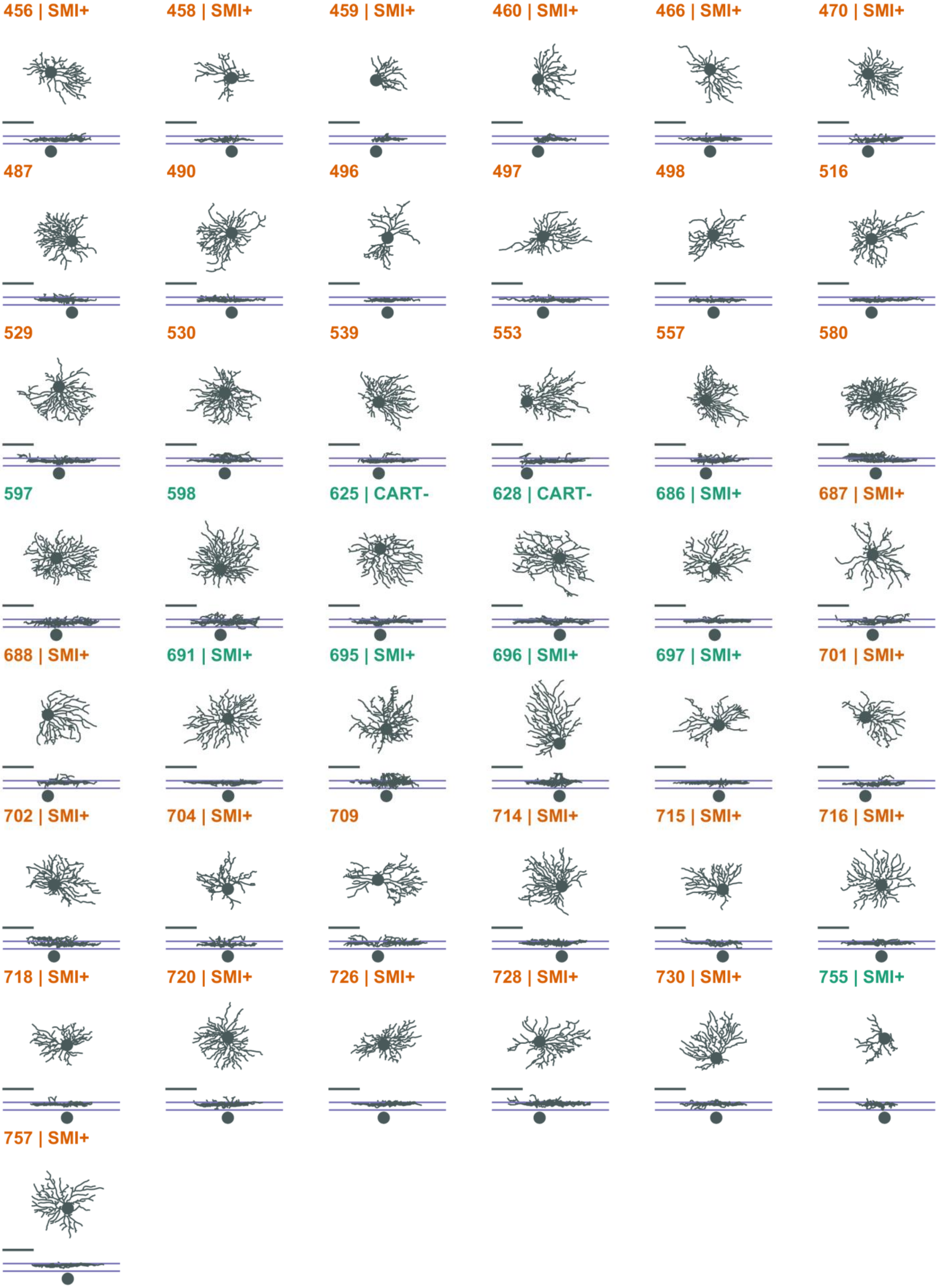
456 | SMI+
458 | SMI+
459 | SMI+
460 | SMI+
466 | SMI+
470 | SMI+
487
490
496
497
498
516
529
530
539
553
557
580
597
598
625 | CART-
628 | CART-
686 | SMI+
687 | SMI+
688 | SMI+
691 | SMI+
695 | SMI+
696 | SMI+
697 | SMI+
701 | SMI+
702 | SMI+
704 | SMI+
709
714 | SMI+
715 | SMI+
716 | SMI+
718 | SMI+
720 | SMI+
726 | SMI+
728 | SMI+
730 | SMI+
755 | SMI+
757 | SMI+

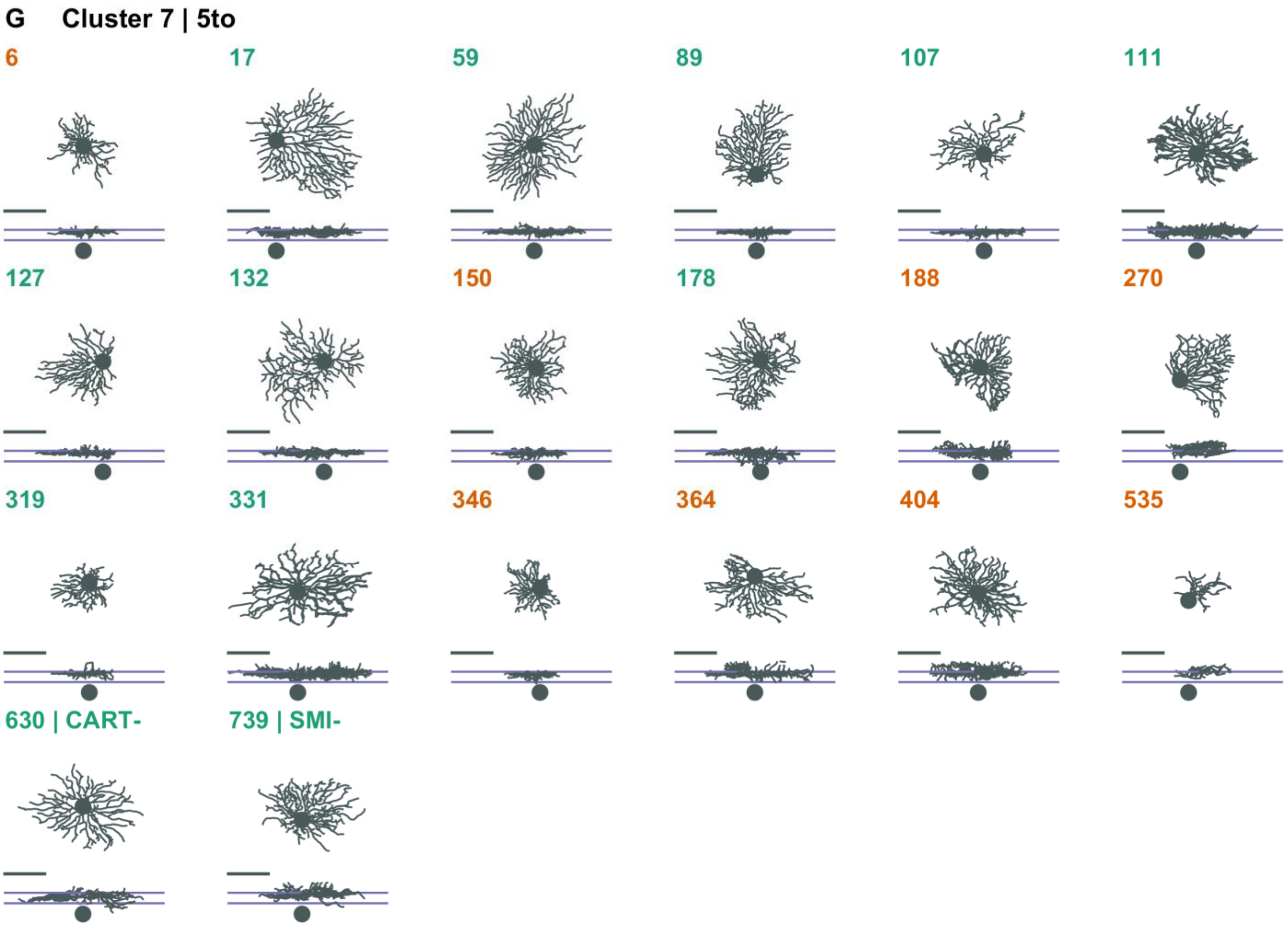
G
Cluster 7 | 5to
6
17
59
89
107
111
127
132
150
178
188
270
319
331
346
364
404
535
630 | CART-
739 | SMI-

H Cluster 8 | 5si | HD1 or HD2

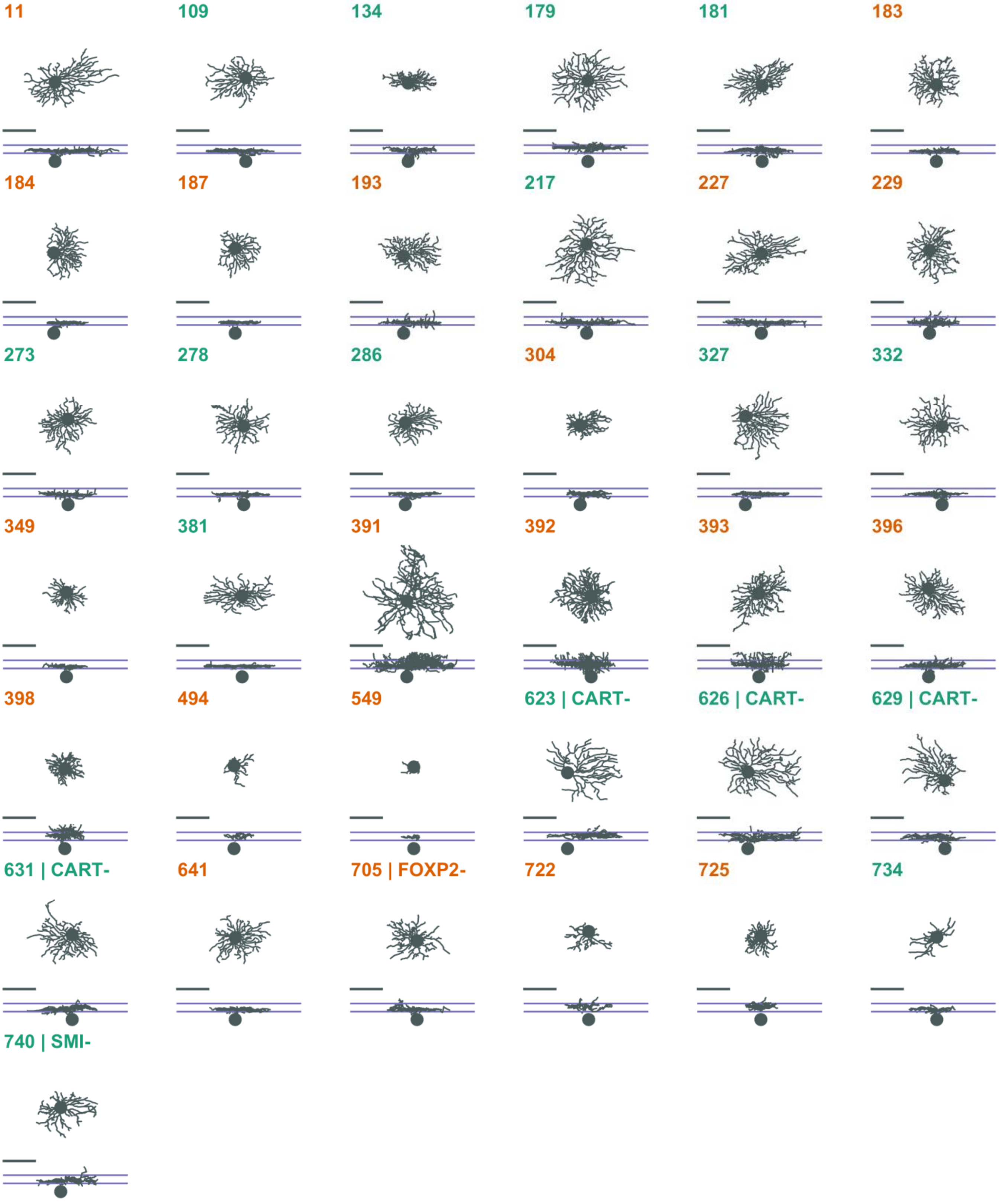

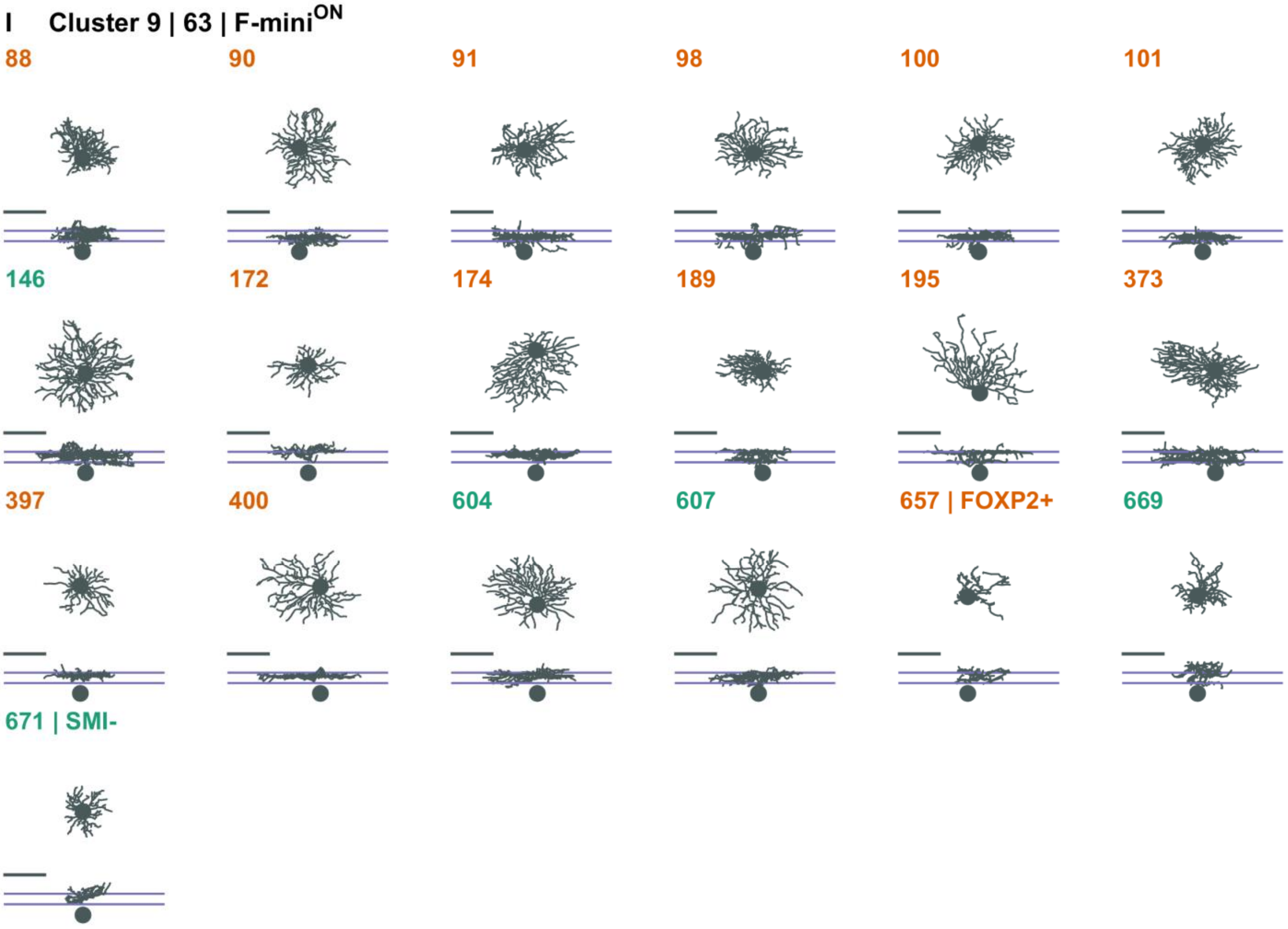

I Cluster 9 | 63 | F-mini^{ON}
88
90
91
98
100
101
146
172
174
189
195
373
397
400
604
607
657 | FOXP2+
669
671 | SMI-

J Cluster 10 | 6sn

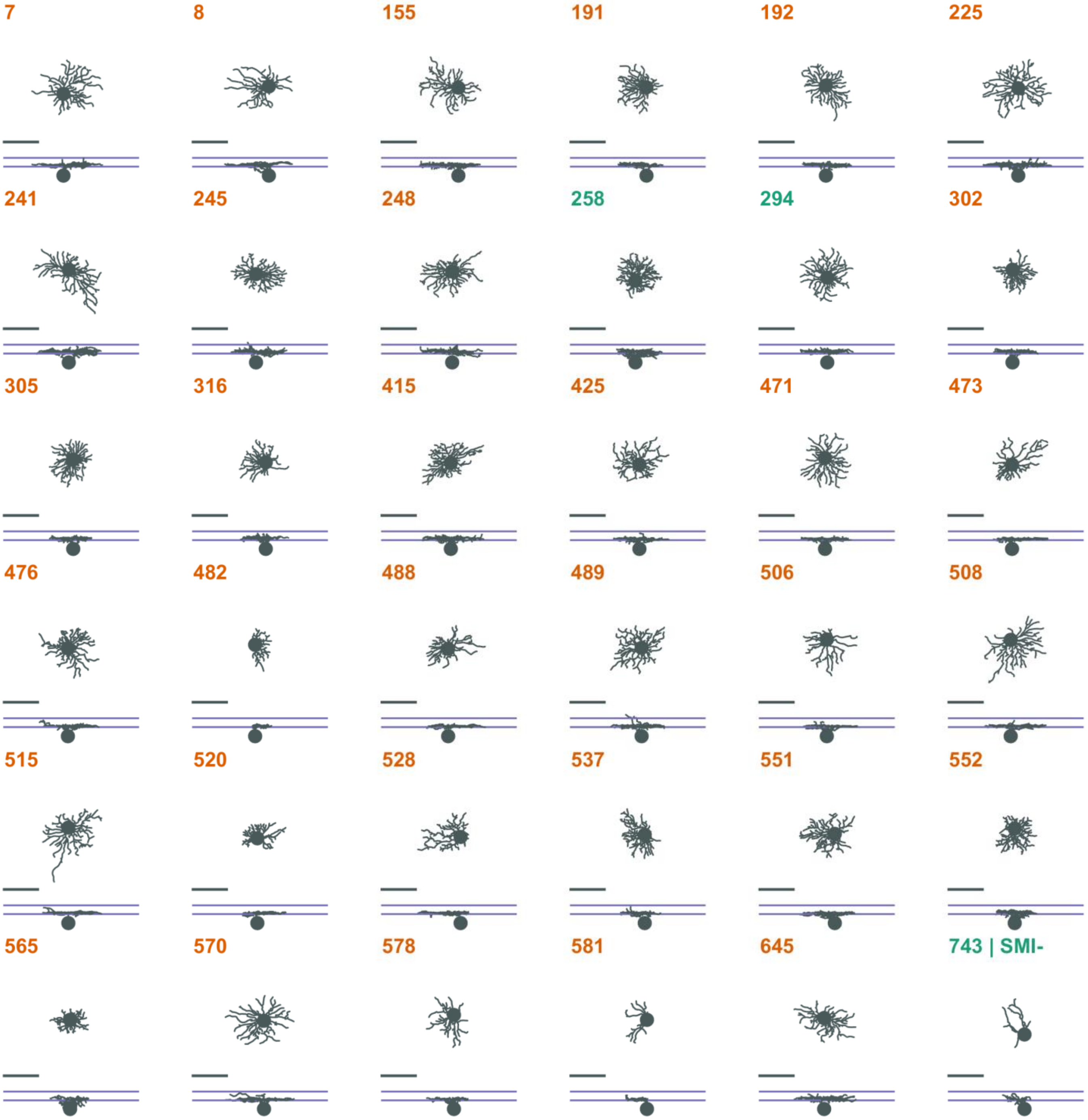

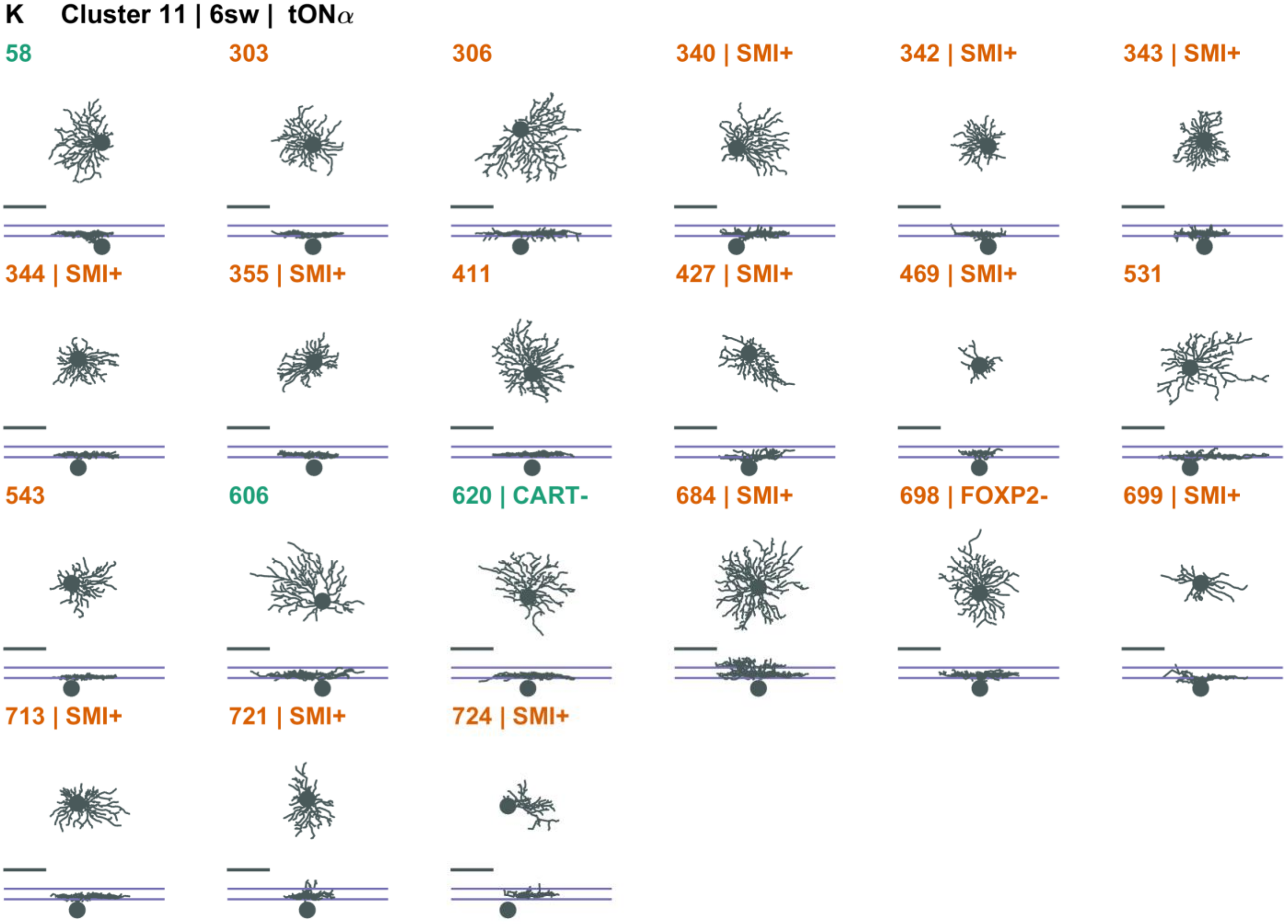
K
Cluster 11 | 6sw | tONα
58
303
306
340 | SMI+
342 | SMI+
343 | SMI+
344 | SMI+
355 | SMI+
411
427 | SMI+
469 | SMI+
531
543
606
620 | CART-
684 | SMI+
698 | FOXP2-
699 | SMI+
713 | SMI+
721 | SMI+
724 | SMI+

L Cluster 12 | 6t | F-midi^{ON}

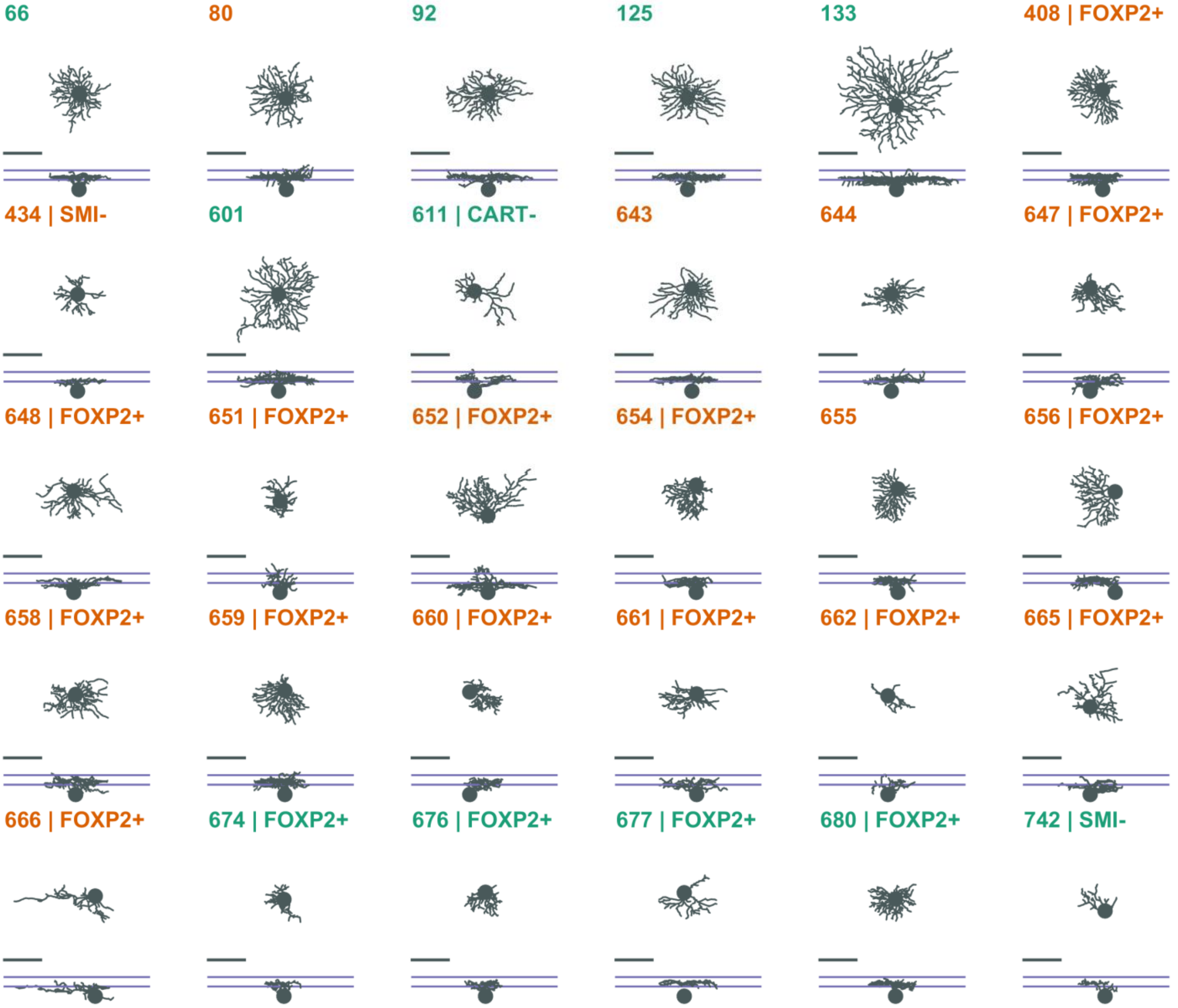

M **Cluster 13 | 8w | sONα**

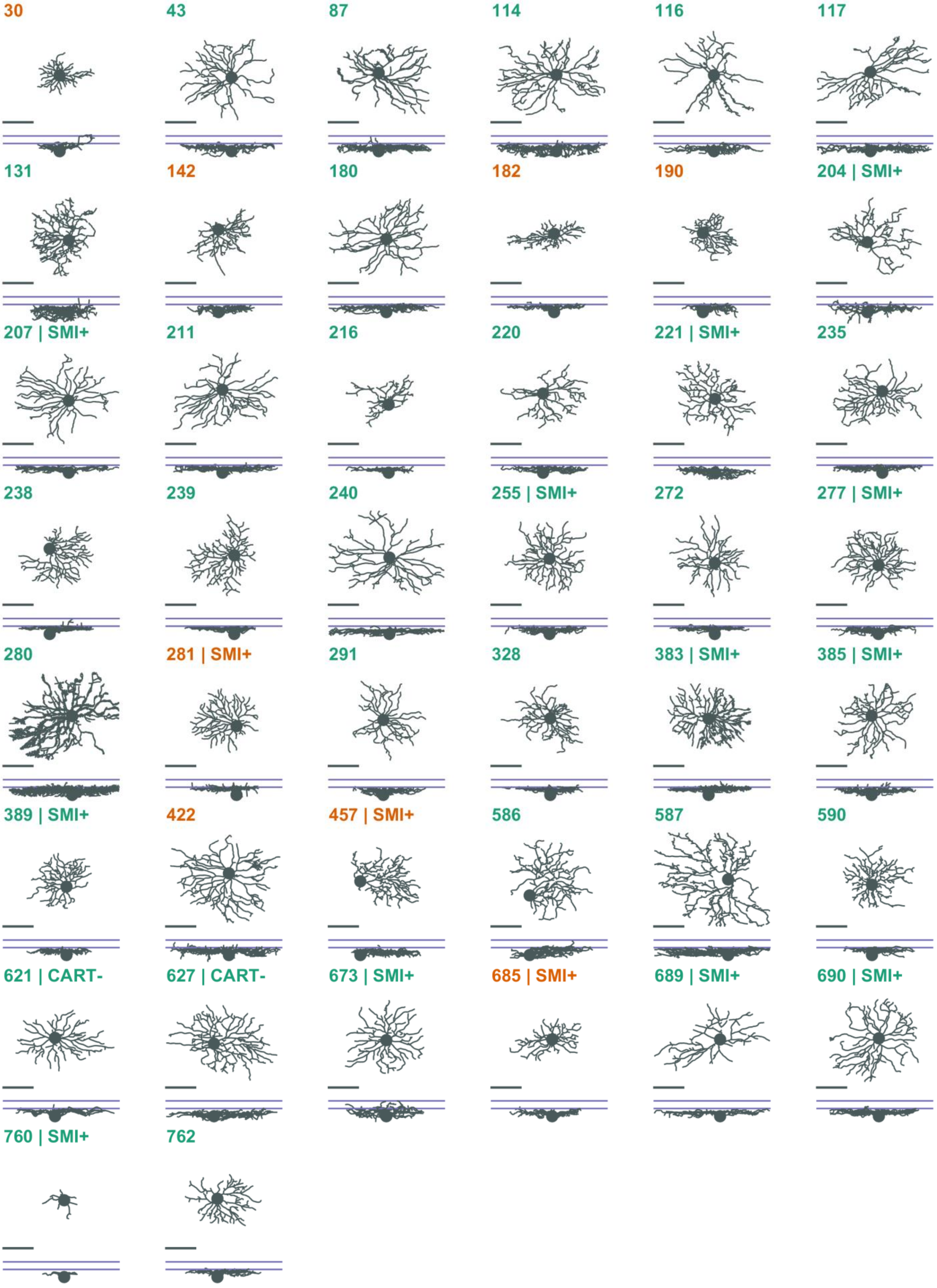

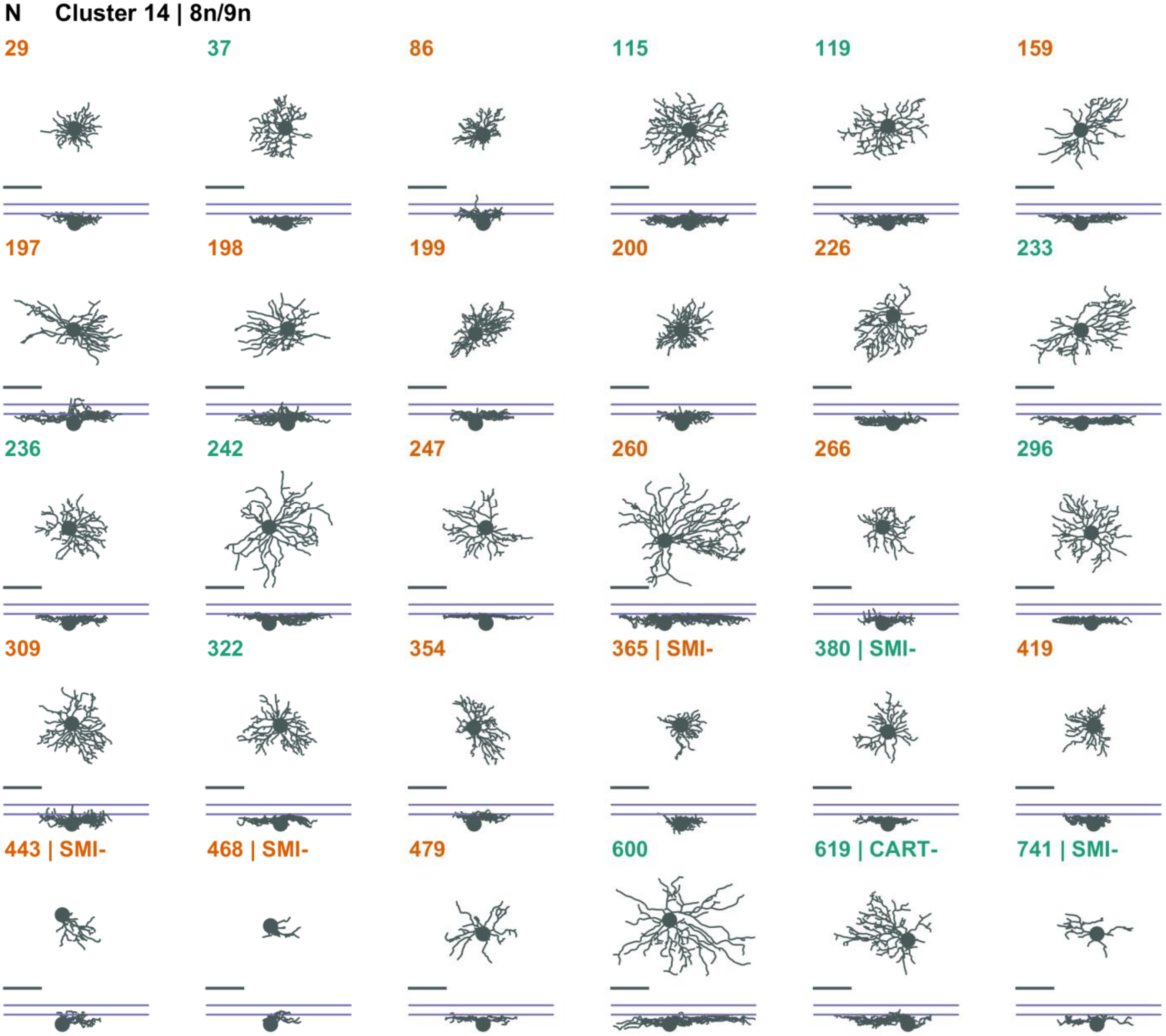

N
Cluster 14 | 8n/9n
29
37
86
115
119
159
197
198
199
200
226
233
236
242
247
260
266
296
309
322
354
365 | SMI-
380 | SMI-
419
443 | SMI-
468 | SMI-
479
600
619 | CART-
741 | SMI-

O Cluster -1 | 1ni | OFF Step

79 151 639 640

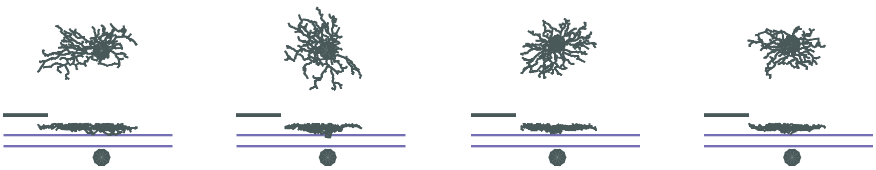

P Cluster -2 | 2o | G5b,c

337

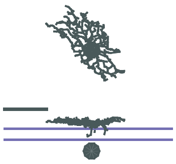

Q Cluster -3 | 25

428 472 554 736

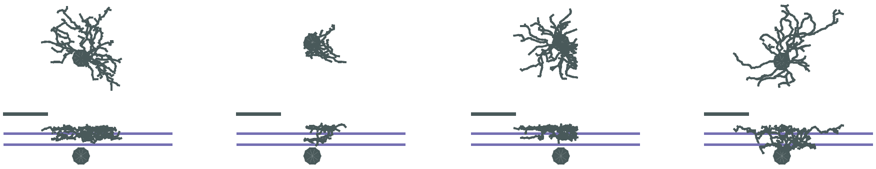

R Cluster -4 | 28

218 394 491 561 584

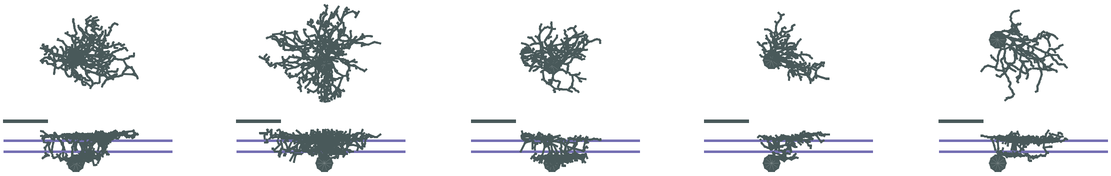

S Cluster -5 | 3i

167 363 703 | SMI-

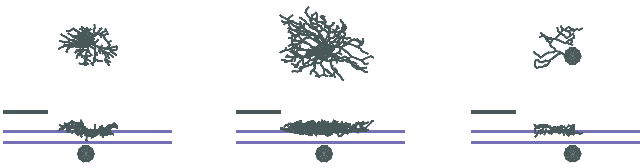

T Cluster -6 | 3o

269

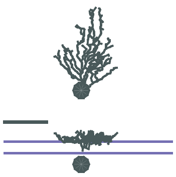

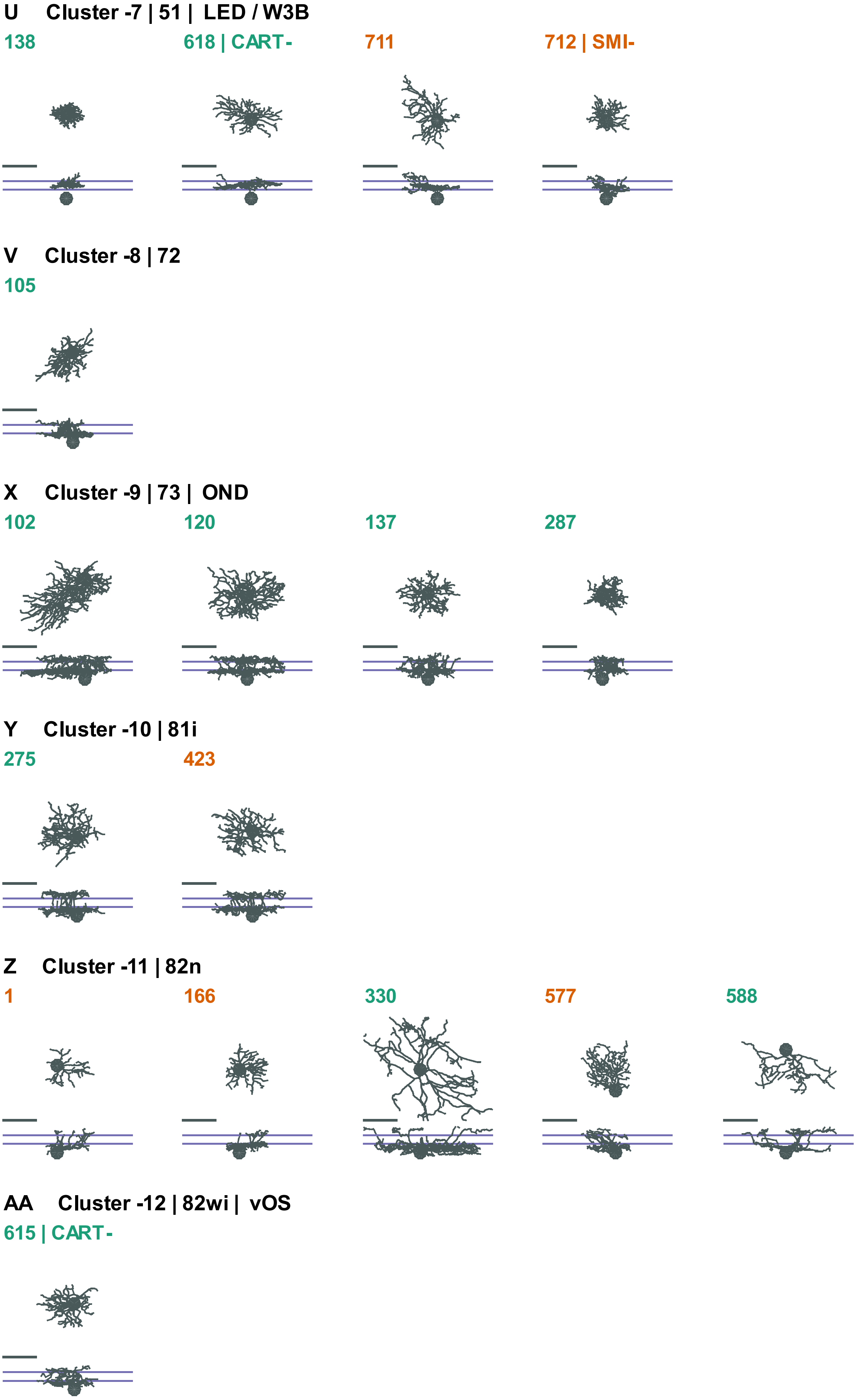
U Cluster -7 | 51 | LED / W3B
138
618 | CART-
711
712 | SMI-
V Cluster -8 | 72
105
X Cluster -9 | 73 | OND
102
120
137
287
Y Cluster -10 | 81i
275
423
Z Cluster -11 | 82n
1
166
330
577
588
AA Cluster -12 | 82wi | vOS
615 | CART-

BB Cluster -13 | 85

536 593 670

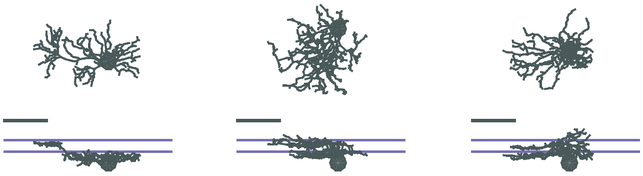

CC Cluster -14 | 9w | M2

633 | CART-

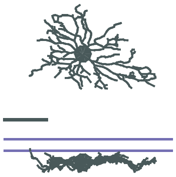

DD Cluster -15 | 91

214 409 692

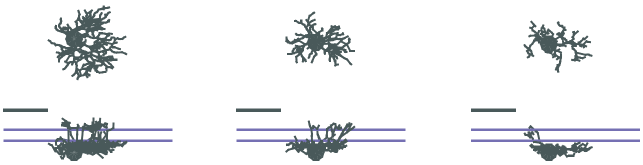

EE Cluster -16 | 915

433 | SMI- 465 | SMI- 745 | SMI-

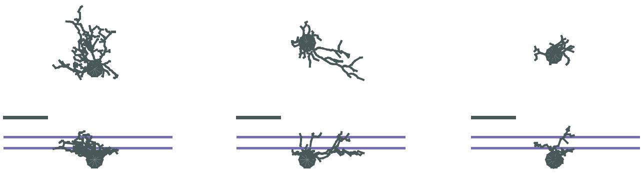

FF Cluster -17 | Fbistrat | F-bistratified

550 558 562 599

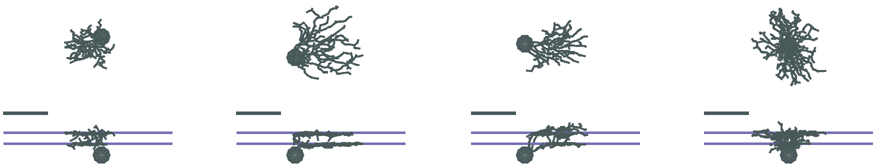

Supplement: Figure 2—source data 1. — Positive cluster numbers are ones included in main text. Negative cluster numbers are clusters assigned less than 1% of the total population. Scale bar: 100 µm. [file elife-50697-fig2-data1.pdf]
